# Covalent–Organic Frameworks for Selective and Sensitive Detection of Antibiotics from Water

**DOI:** 10.3390/polym16162319

**Published:** 2024-08-16

**Authors:** Ying Hao, Yanjie Xia, Jingjing Huang, Chenglin Zhong, Guizhen Li

**Affiliations:** School of Chemistry and Chemical Engineering, Linyi University, Linyi 276005, China; haoying0607@163.com (Y.H.); xiayanjie1004@163.com (Y.X.); 220703001535@lyu.edu.cn (J.H.)

**Keywords:** covalent–organic frameworks, antibiotics, detection, application

## Abstract

As the consumption of antibiotics rises, they have generated some negative impacts on organisms and the environment because they are often unable to be effectively degraded, and seeking effective detection methods is currently a challenge. Covalent–organic frameworks (COFs) are new types of crystalline porous crystals created based on the strong covalent interactions between blocked monomers, and COFs demonstrate great potential in the detection of antibiotics from aqueous solutions because of their large surface area, adjustable porosity, recyclability, and predictable structure. This review aims to present state-of-the-art insights into COFs (properties, classification, synthesis methods, and functionalization). The key mechanisms for the detection of antibiotics and the application performance of COFs in the detection of antibiotics from water are also discussed, followed by the challenges and opportunities for COFs in future research.

## 1. Introduction

Antibiotics were first discovered by A. Fleming in 1929, and since then, large quantities of antibiotics have been widely used in aquaculture, the livestock industry, and medical treatment [1]. Antibiotics refer to a class of secondary metabolites produced by microorganisms (including bacteria, fungi, and actinomycetes) or higher animals and plants in the process of life, which have anti-pathogen or other activities and can interfere with the development of other living cells [2]. The abuse of antibiotics results in a negative influence on the environment and human health, including toxic effects, reproductive disorders, and disruption of the ecological balance [3]. Receptors are found to be synergistic to antibiotics or their residues and result in severe diseases, even in very low concentrations. Moreover, one key issue is that antibiotics are highly cumulative and not easy to degrade, resulting in biological effects [4].

Thus, many countries strictly regulate the use of antibiotics and limit the maximum residue of antibiotics in different products [5]. Therefore, it is of great importance to develop sensitive and selective methods for the detection of antibiotics, especially in large antibiotic abuse settings [6]. Currently, for antibiotic identification in water environments, many methods have been established, including high-performance liquid chromatography (HPLC), gas chromatography (GC), liquid chromatography–mass spectrometry (LC-MS), surface-enhanced Raman spectroscopy (SERS), and so on [7,8]. However, these traditional methods expose many shortcomings, such as high costs, long use times, and possible secondary pollution caused by the intermediates [9]. Therefore, finding efficient, energy-saving and effective materials and methods is a key task for the detection of antibiotics.

**Figure 1:**
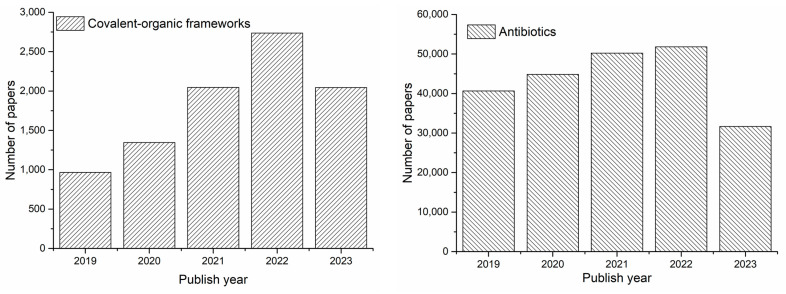
Number of papers about covalent–organic frameworks and antibiotics published since 2019. These papers were obtained from the Science Citation Index Expanded (SCIE) database of the Institute for Scientific Information (ISI), searched using “Covalent-organic frameworks, and antibiotics” as titles.

Covalent–organic frameworks (COFs) were first discovered by the Yaghi group in 2005 [10]. Their organic building units are connected by covalent bonds to form a porous skeleton with a periodic structure through dynamic covalent bonds of light elements (B, C, N, O, and Si), thus having excellent properties [11]. The covalent bonding linkage of COFs mainly includes a boric acid ester bond, an imine-based linkage, a triazine-based linkage, and so on [12]. According to its spatial structure, COFs can be divided into two-dimensional (2D) and three-dimensional (3D) structures [13]. In 3D COFs, the organic units are connected by covalent bonds to form a three-dimensional network structure, and the 3D structure material is widely used in catalysis, gas adsorption, and other aspects [14]. In 2D COFs, organic units are connected with two-dimensional atomic layers, and atomic layers are further stacked to form layered structures through π–π interaction, and the whole frame structure is determined by covalent bonds within layers and controlled by non-covalent forces between layers, so it has a broad application prospect in the energy storage field [15]. Because of their unique advantages, such as high thermal stability, large specific surface area, rich pores, adjustable molecular structure, and many active sites, COFs have been gradually applied to the fields of catalysis, separation, photoelectricity, energy, and so on [16,17]. Figure 1 shows the number of papers about COFs and antibiotics published since 2019.

In recent years, various materials, like metal–organic frameworks (MOFs), carbon nanomaterials, nanoparticle biosensors, and molecularly imprinted polymers (MIPs), have been designed and developed for the detection of antibiotics [18]. However, these materials still have the drawbacks of expensive costs, toxic residues, complicated pretreatment processes, and so on [19]. Therefore, convenient and low-cost detection materials with high accuracy and sensitivity are desirable [20]. In comparison with these materials, COFs have higher porosity and bigger and tunable pore sizes, which speed up the diffusion of reactants and desorption of products, thereby promoting higher yield and selectivity [21]. Moreover, some excellent reviews have been published about COFs in the detection of pharmaceuticals, and in this way, there is a great need and room for the analysis of COFs in antibiotic detection [22].

This review first aims to present state-of-the-art insights into COFs (properties, classification, synthesis methods, and functionalization). Secondly, the key mechanisms for the detection of antibiotics and the application performance of COFs in the detection of antibiotics from water are also discussed. Finally, the challenges and opportunities of COFs in future research are also discussed.

## 2. Antibiotics

### 2.1. Classification and Synthetic Pathways of Antibiotics

According to the different classification standards, antibiotics can be classified as the following:

Firstly, according to the chemical structures, antibiotics can be divided into quinolone antibiotics, β-Lactam antibiotics, macrolides, aminoglycoside antibiotics, etc. [23].

Secondly, according to the function, antibiotics can be divided into antibacterial antibiotics, antifungal antibiotics, anti-tumor antibiotics, antiviral antibiotics, livestock antibiotics, agricultural antibiotics, and others [24].

Antibiotics can be produced in various ways according to their types, such as penicillin biosynthesis through microbial fermentation and sulfonamides, quinolones, etc., through chemical synthesis [25]. There are also semi-synthetic antibiotics, and they are derivatives made by modifying the molecular structure of antibiotics obtained through biosynthesis using chemicals (biological or biochemical methods) [26].

### 2.2. Hazards of Antibiotics

There are five major hazards of antibiotics, including intestinal microbiota imbalance, fungal infections, leukopenia, liver and kidney dysfunction, and gastrointestinal symptoms [27]. Firstly, antibiotics not only kill harmful bacteria but also kill normal gut microbiota, which can cause dysbiosis of gut microbiota and lead to diarrhea, known as antibiotic-related diarrhea [28]. Secondly, if there is long-term use of antibiotics, a decrease in the normal bacterial population in the body and fungal reproduction can be easily caused, resulting in fungal enteritis, thrush, etc. [29]. Thirdly, the use of antibiotics can also cause damage to normal cells in the body, leading to a decrease in neutrophils and white blood cells [30]. Fourthly, the use of antibiotics for a long time and in large doses can easily cause drug-induced hepatitis, liver function damage, etc., and drug excretion through the kidneys can also increase the burden on the kidneys [31]. Fifthly, the use of antibiotics may result in gastrointestinal symptoms, such as diarrhea, abdominal pain, nausea, and vomiting [32].

## 3. COF Structure and Classification

### 3.1. COF Structure

COFs are another important three-dimensionally ordered material emerging after metal–organic framework materials (MOFs) [33]. There are many important factors in determining the geometries of COFs, such as size, morphology, structure, and the dynamic nature of the bonds used to connect components [34]. COFs are composed of strong covalent bonds (C-C, C-O, C-B, and B-O), and they have high thermal stability, high surface area, and extremely low density [35]. In addition to their well-organized structure, COFs can also be designed with holes of their size and chemical characteristics according to the selection of initial structural units and adjusting its side chain length [36].

**Figure 2:**
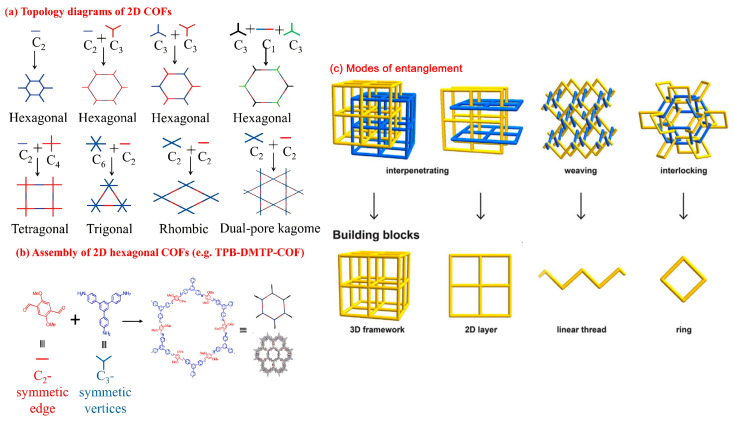
(**a**) Topology diagrams of 2D COFs; (**b**) assembly of 2D hexagonal COFs; (**c**) COFs’ four modes of entanglement in frameworks. Reproduced with permissions from Ref. [37], Elsevier, copyright 2019, and Ref. [39], The American Association for the Advancement of Science, copyright 2017.

COFs can be divided into 2D and 3D COFs according to the topological structure [37,38] (Figure 2). In 2D COFs, monomers are connected by covalent bonds to form a layered structure in the plane. Conjugate systems are formed between layers through π–π interaction, and one-dimensional channels are also formed. The size and shape of the channels are closely related to the stacking mode between layers [39]. The high selectivity and permeability of 2D COFs render them with high separation performance and are then successfully utilized for purification applications [40]. For 3D COFs, the monomer extends infinitely through covalent bonds to form a framework with regular and periodic structure [41]. Different from 2D COFs, 3D COFs can easily form large, cage-like cavities when they are extended in 3D space, while monomers can continue to react in the cavities and grow outwards so that 3D COFs often have multiple interpenetrating structures, and 3D COFs with enhanced reactant and active sites accessibility exhibit potential for separation due to their large specific surface area and high porosity [42].

### 3.2. COF Classification

**Figure 3:**
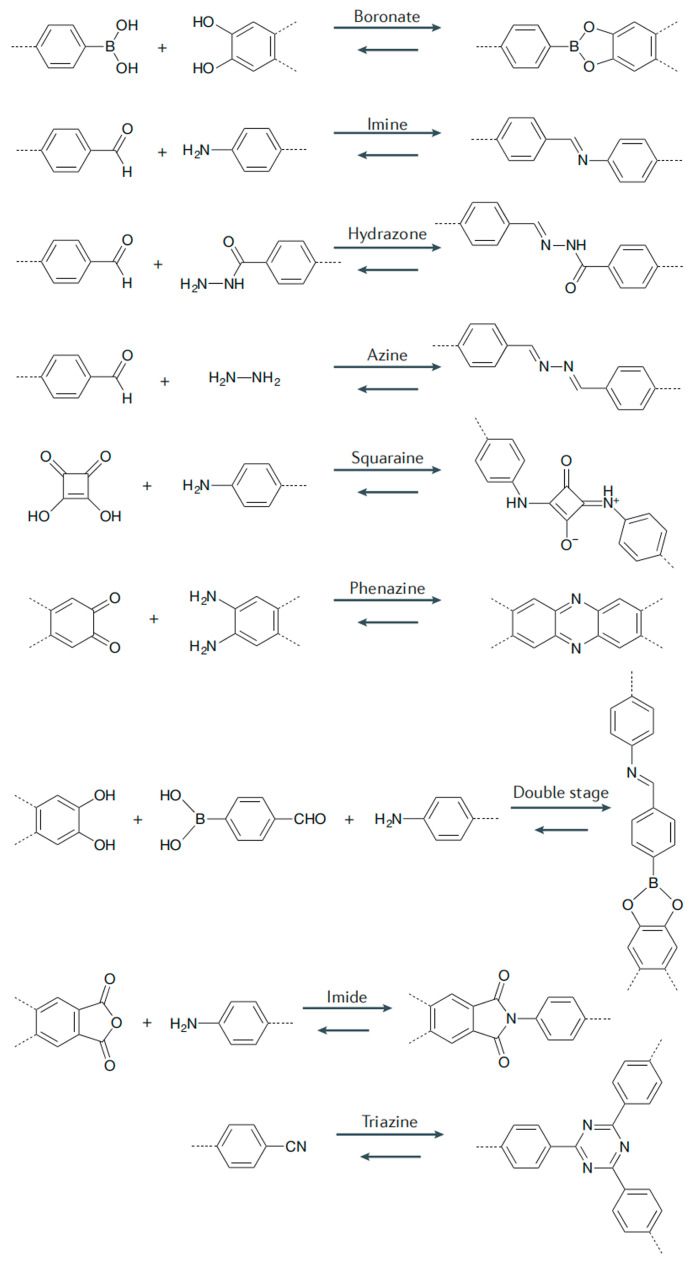
Diversity of linkages for the formation of COFs. Reproduced with permissions from Ref. [43]; Springer Nature, copyright 2016.

In general, COFs can be divided into boron, triazine, imine, phenyl hydrazone, ketone enamine, polyimide, phthalocyanine, and porphyrin according to the type of covalent bond formed by polycondensation [43]. Figure 3 shows the diversity of linkages for the formation of COFs. The covalent bond connecting COFs endows them with high-temperature resistance, but the other chemical stability of different covalent bonds of COFs is different [44]. As boroxines and boronic esters are water-labile bonds and rather sensitive to hydrolysis, the introduction of the imine linkage formed by the condensation between aldehydes and primary amines supports the development of more stable coupling motifs, as well as with hydrazones, azines, and imides [45]. Moreover, β-ketoenamines with further improved chemical stability forming from primary amines and 1,3,5-triformylphloroglucinol undergo a second and irreversible enol-keto-tautomerization to create networks that are stable against acids and bases [46].

#### 3.2.1. Boron-Based COFs

The first boron-based COFs (COF-1 and COF-5) were prepared by Yaghi and coworkers, and since then, diverse boron-based COFs have been synthesized through the formation of boronate ester, boroxine, or borazine [47]. According to the synthetic strategies, most boron-based COFs can be clarified into two categories: single building block self-condensation and two or more building unit co-condensation [48]. Yaghi et al. first synthesized boron-based COF-1 with 1,4-benzene diboronic acid at 120 °C, and the obtained COF-1 had a layered structure of a hexagonal graphene-like structure formed by the interconnection of B_3_O_3_ rings [49]. Liu et al. used this reaction to obtain COF-102 and COF-103, and they were boron-containing polymers with a C-C bond and Si-C bond as the central connection points, respectively [50]. To improve the stability of boron-based COFs, Li et al. introduced anionic boron centers fabricated of a spiroborate-linked COF, slowing down its hydrolysis and improving its structural controllability while improving the stability of COFs. The research on COFs was also promoted to a new height [51].

Boron-based COFs generally possess low density, a high surface area, and high thermal stability, leading to various applications, but most of these materials have the defect of easy decomposition in water due to the electronic deficiency of the boron element. They are often unstable in a humid environment, and some of them may undergo structural transformation even under air [52].

#### 3.2.2. Triazine-Based COFs

The first triazine-based COFs (donated as CTFs-1) were achieved by Thomas and co-workers in 2008 through cyclotrimerization of aromatic nitriles at 400 °C [53]. At present, only a few triazine-based COFs have been successfully synthesized using this method because the reaction conditions are harsh, the synthesis process is difficult to control, and the requirements for the thermal stability of the precursor are high, and it is not conducive to mass production [54]. Thus, other strategies based on milder conditions have been developed, and a low-temperature polycondensation approach was utilized to synthesize CTFs based on a broader range of building blocks via mild conditions [55].

Triazine-based COFs have many advantages, such as a high surface area and remarkable thermal and chemical stabilities. In the study by Li, L. et al., the stability of the triazine-based COFs could retain 88% of the initial capacitance after 20,000 charge−discharge cycles in the cycle life test, and such excellent cycle stability has rarely been reported in analogous materials [56]. Wang et al. showed that triazine-based COFs had a surface area of 789 m^2^ g^−1^, a total pore volume of 0.37 cm^3^ g^−1^, and a pore size of 1.23 nm because of the triazine framework. Their high specific area and specific active sites made them excellent carriers, which had broad application prospects in separation, analysis, photoelectric, heterogeneous catalysis, electrochemistry, and other fields [57].

#### 3.2.3. Imine-Based COFs

Imine-based COFs are another representative class of COFs, which are based on the fact that the formation of an imine is a reversible reaction [58]. At present, the synthesized imine-based COFs mainly include two groups: one group is formed via the co-condensation of aldehydes and amines, and the other group is formed by the co-condensation reaction of aldehydes and hydrazides [59]. The first imine-based COF (COF-300) was developed by Yaghi and co-workers through the reaction of aldehyde and amine [60]. Since then, F. J. Uribe Romo et al. successfully prepared 3D COF-300 with good thermal stability and chemical stability and connected by an imine bond using the amine aldehyde condensation method for the first time [61]. Yan and co-workers achieved imine-based COFs with 5.3 nm wide pore loadings with Rhodamine B. Rhodamine B’s luminescence intensity was inversely proportional to temperature, and it also showed good optical sensing characteristics [62]. Jiang et al. demonstrated imine-based COFs loaded with N-heterocyclic compounds (triazole and imidazole) with high proton conductivity 2–4 orders of magnitude higher than nonporous or microporous systems [63].

Imine-based COFs have attracted large interest in many areas because they combine the advantages of boron-containing COFs and triazine-based COFs, for example, much better crystallinity and structural regularity than triazine-based COFs and better chemical stability in most organic solvents or water compared with boron-containing COFs [64].

#### 3.2.4. Hydrazone-Based COFs

Hydrazone-based COFs are connected by hydrazone bonds formed by the dehydration and condensation of aldehyde and hydrazine and were first studied by Uribe Romo et al. [65]. Hydrazone-based COFs have good stability for hydrolysis, which is conducive to the selective resolution of heteroporous COFs due to the stability of hydrazone holding units [66]. Hydrazone-based COFs have been widely used in the field of photocatalysis, separation, and electrochemistry due to their unique photoelectric properties, good light absorption ability, and physical and chemical stability [67].

#### 3.2.5. Azine-Based COFs

The first azine-linked COFs were synthesized by the condensation of 1,3,5,8-tetrakis(4-formylphenyl)pyrene with hydrazine, and for azine linkage, hydrazine acted as a common building block to react with diverse aldehydes [68]. Azine-based COFs could provide abundant active sites for specific guest molecules; in addition, nitrogen-containing COFs had directional and vertical one-dimensional channels and an orderly periodic network structure, which enabled small guest molecules to diffuse smoothly in the channels without blocking. Therefore, they had great advantages in separation, adsorption, and the removal of harmful molecules [69].

#### 3.2.6. β-Ketoenamine-Based COFs

β-ketoenamine-based COFs have keto-enol tautomerism; for example, Banerjee et al. prepared a kind of β-ketoenamine-based COF through irreversible enol–keto tautomerization of ketenimine linkages in imine-based COFs [70]. β-ketoenamine-based COFs have excellent adsorption performance and catalytic activity, and at the same time, the forward reaction rate is fast, which leads to the self-repair and insufficient reassembly ability of such polymers, and it becomes very difficult to form an ordered structure [71].

#### 3.2.7. Imide-Based COFs

Imide-based COFs were first reported by Yan’s group in 2014. They were fabricated via a reversible imidization reaction and simply by extending building molecules, and the large pore size of imide-based COFs could be tuned [72]. The imide-based COFs exhibited a high surface area and had great potential for loading dye molecules and drug delivery [73].

## 4. Synthetic Methods of COFs

**Figure 4:**
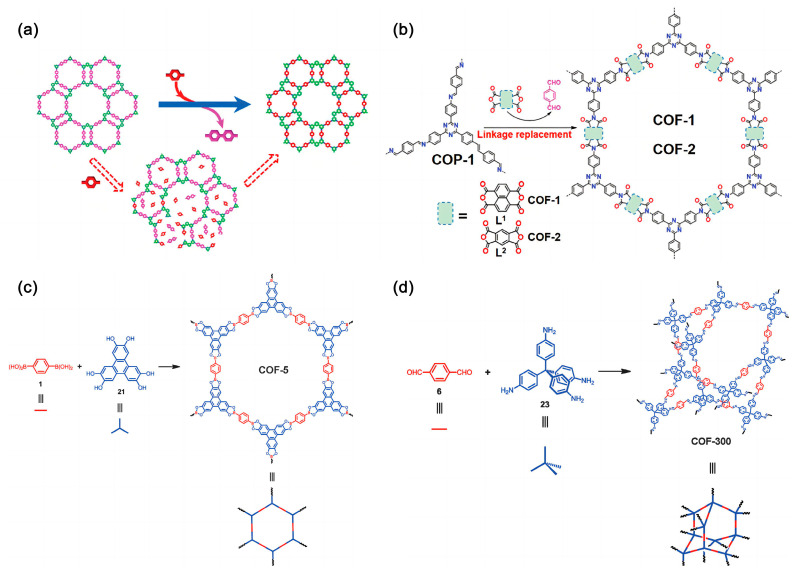
The proposed process for synthesis of COFs. (**a**) Proposed process for the in situ transformation of TP–COF–BZ into TP–COF–DAB in the presence of 1,4-diaminobenzene. (**b**) Conversion of amorphous COP-1 to COFs using the linker-exchange strategy. (**c**) Assembly of 2D hexagonal COFs (COF-5 as an example) from linear and trigonal building units. (**d**) Assembly of 3D COFs (COF-300 as an example) from linear and tetrahedrally-structured building units. Reproduced with permissions from Ref. [75], ROYAL SOCIETY OF CHEMISTRY, copyright 1972.

The development of simple and effective synthesis methods is very important for the large-scale application of COFs [74]. Figure 4 shows the proposed process for the synthesis of COFs. The main synthesis methods include ionic heating, microwave-assisted solvent heating, mechanical grinding, and solvothermal methods [75,76].

The thermodynamic reversible reaction is conducive to the formation of a long-range ordered structure, so COFs are generally prepared by a thermodynamic reversible reaction [77]. A single or mixed solvent is used to dissolve some of the monomers, and COF microcrystalline aggregates are obtained by long-term reaction under high-temperature and low-pressure conditions. During the process, polymerization, crystallization, assembly, and other processes are involved [78]. The synthesis of COFs is affected by the reaction temperature, time, composition, etc. In the synthesis process, their morphology, size, surface properties, and functions can be controlled [79].

### 4.1. Ionothermal Synthesis

**Figure 5:**
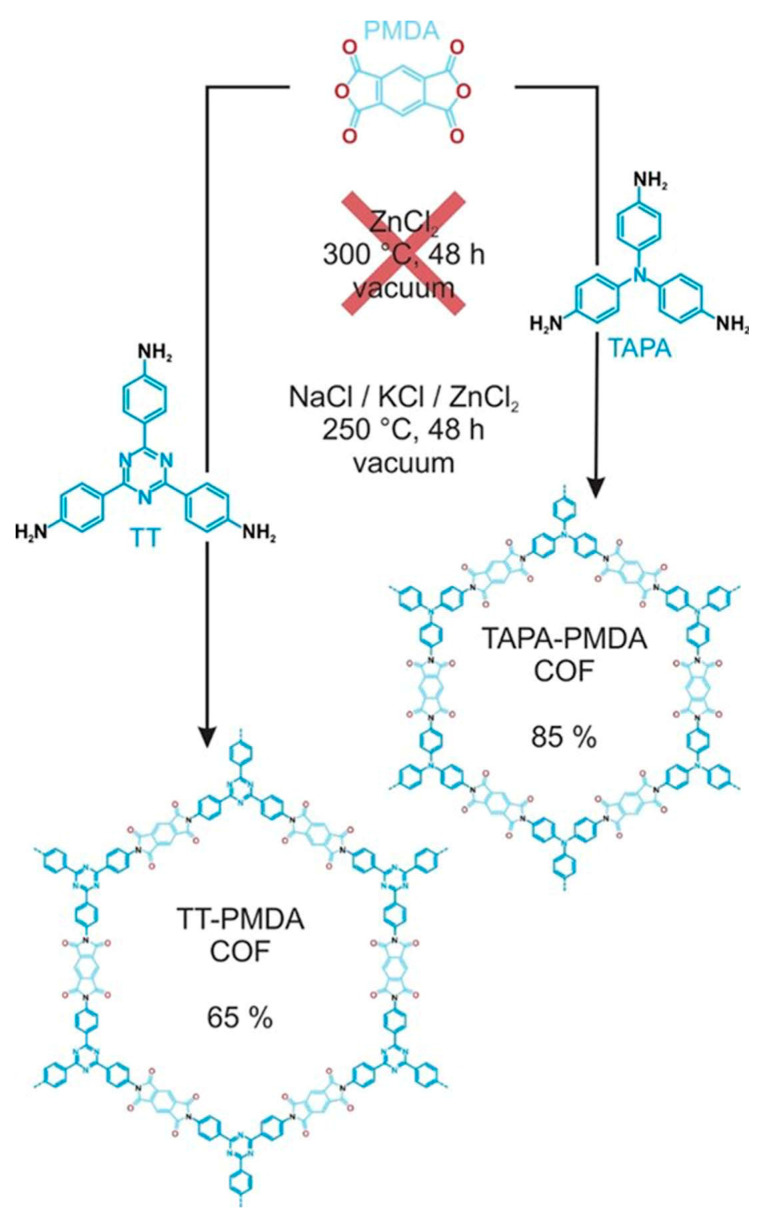
Synthesis of COFs using ionothermal method. Reproduced with permissions from Ref. [80]; Wiley Online Library.

So far, the ionothermal synthesis of COFs has only been used for the synthesis of triazine COFs [80]. Figure 5 shows the synthesis of COFs using the ionothermal method.

Thomas’s research group first synthesized CTF-1 COF material using the ionothermal method at 400 °C using molten ZnCl_2_ as the solvent. After 40 h, when the system temperature dropped to room temperature, the amperometric flask was opened to obtain CTF-1 after a series of solid treatments [81]. Due to the strict reaction conditions and high reaction temperature of this method, the requirements for thermal stability of the constituent units are also increased, and at present, the use of ionothermal synthesis of COFs is relatively rare [82].

### 4.2. Microwave-Assisted Synthesis

**Figure 6:**
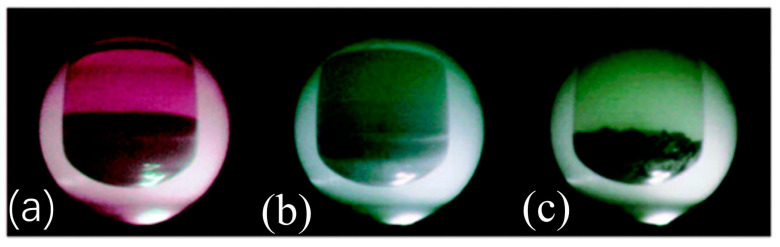
Images of COF-5 using microwave-assisted method (gray COF-5 with purple impurities (**a**), removal of impurities via MW extraction process (**b**), and purified gray COF-5(**c**)). Reproduced with permission from Ref. [85], Royal Society of Chemistry, copyright 2014.

The microwave-assisted synthesis method refers to the use of microwave heating, and compared with the traditional heating method, it does not need a heat-conduction process and can achieve uniform heating in a short time [83]. Microwave-assisted synthesis was first used to synthesize boron-based COFs in 2009. Compared with traditional solvothermal methods, microwave-assisted synthesis can be realized in a short time (within 20 min), with a higher specific surface area (2019 m^2^ g^−1^ for COF-5 and 2926 m^2^ g^−1^ for COF-102). The development of the microwave-assisted method provides a simple, rapid, and efficient method for the synthesis of stable COFs, which is expected to be a promising method for the industrial production of COFs [84]. Cooper’s group synthesized 2D COF-5 and 3D COF-102 via a rapid microwave synthesis method in 20 min with higher surface areas compared to other synthesis methods [85]. Figure 6 shows the images of COF-5 using the microwave-assisted method (gray COF-5 with purple impurities (a), the removal of impurities via the MW extraction process (b), and purified gray COF-5 (c)).

The main characteristics of microwave heating are fast heating speed, energy saving, high efficiency, easy control, etc. Therefore, using microwave heating can improve the reaction speed and shorten the reaction time, which is a very promising overall heating method for COF synthesis [86].

### 4.3. Room-Temperature Synthesis

Room-temperature syntheses chiefly consist of two techniques: mechanochemical grinding and the solvothermal method [87].

Compared with the traditional solvothermal method, the mechanical grinding synthesis of COFs has the characteristics of simple operation, fast speed, environmentally friendly, and high production capacity [88]. Biswal and co-workers synthesized 1,3,5-triformylphlorog lucinol-diamine COFs using the room-temperature synthesis method [89]. However, due to its low productivity and general performance, there are few examples of this method being used to synthesize COFs [90].

### 4.4. Solvothermal Method

**Figure 7:**
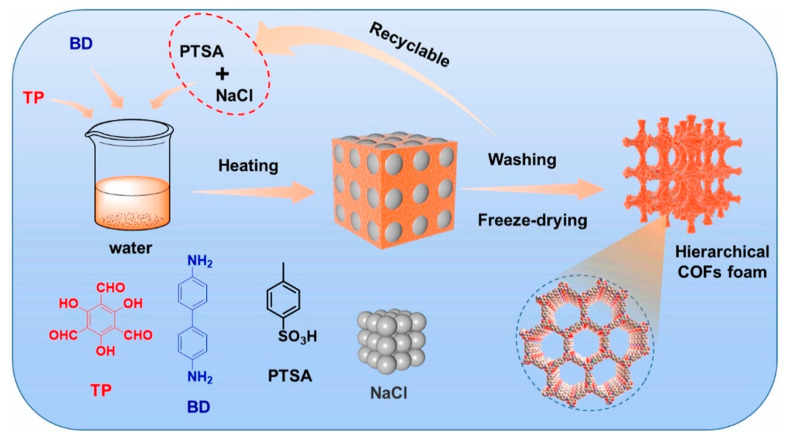
Preparation of COFs via solvothermal synthesis approach. Reproduced with permissions from Ref. [92]; Elsevier, copyright 2022.

The solvothermal method is a commonly used method to synthesize COFs; for example, Shiraki et al. reported the synthesis of COFs in 1,4-phenylenediamine and 1,3,5-benzenetricarbaldehyde using the solvothermal method [91]. Matsumoto et al. established a solvothermal method synthesis of 2D imine COFs with high stability and BET surface areas (2175 m^2^ g^−1^) [92]. Figure 7 shows the preparation of COFs via the solvothermal synthesis approach. The solvothermal synthesis method refers to the reaction of mixtures with organic or non-aqueous solutions as solvents under a certain temperature and pressure [93]. The general synthesis step is to add monomer and solvent into the Pyrex tube, freeze with liquid nitrogen, vacuum, thaw, repeat the above operation three times, and remove oxygen from the system. Then, seal the Pyrex tube with a flame lance, put it into a thermostatic oven, and after several reactions, a large amount of insoluble substances will be produced in the system. Finally, COFs can be obtained through a series of purifications [94].

## 5. Physical and Chemical Properties of COFs

### 5.1. Chemical and Thermal Stability

The chemical and thermal stability of COFs are significant features that distinguish them from other materials [95]. Firstly, the strong stability of COFs is determined by its constituent monomers. The monomer elements include linkages, knots, edges, hydrazone, imine, and azine, as well as some monomers with electron-donating groups (such as methoxy, hydroxyl, and amino), and these groups are usually introduced into the phenyl edge to enhance the chemical stability of COFs [96]. Secondly, the stability is related to the strong covalent bond connecting the mediating elements that form a highly sustainable structure, such as the Ce-N bond, Be-O bond, and intermolecular hydrogen bond, which can improve the stability of COFs [97]. Thirdly, they are also related to the synthesis conditions and methods, such as polycondensation, photocatalysis, and synthesis in a closed heat-resistant tube under solvothermal conditions, to ensure that H_2_O can be used for bond self-correction and increase their chemical and thermal stability [98].

### 5.2. High Porosity and Large Specific Surface Area

The pore size of COFs is directly affected by the length of the organic functional groups, and the longer the organic ligand is, the larger the pore size of the material after removing the guest molecules [99]. The COFs have the properties of high porosity, relatively small pore size, and adjustable pore size, which are conducive to rapid molecular diffusion and transport and show excellent properties in practical applications [100].

The specific surface area is another important index used to evaluate the catalytic performance and adsorption capacity of porous materials [101]. By continuously changing the monomers and covalent bonds of COFs, the specific surface area of COFs can be increased. Most of the surface areas of COFs reported in the relevant literature exceeded 1000 m^2^/g, and the specific surface areas of COF-102 and COF-103 reached 3472 m^2^/g and 4210 m^2^/g [102].

COFs are entirely built from organic building blocks connected by covalent bonds having π-conjugated structures, and many COFs have high sustainability, chemical stability, and modest electronic properties for the detection of antibiotics [103]. The properties of different kinds of reported COFs are tabulated in Table 1.

### 5.3. Rigid Topology and Good Modifiability

The topological structure of COFs extends from zero dimensions to three dimensions, and its species extend from amorphous to crystalline [104]. Dynamic covalent chemistry is the scientific basis for the directional design of COFs, and in the process of covalent assembly, it has a high degree of adjustable deformation and is also the key to obtaining crystallinity and stability [105].

There are many adjustable measures for COFs, which can not only selectively introduce functional groups but can also take inorganic particles, such as molecular sieve, activated carbon, carbon nanotubes, and porous silica, with electron pairs as active centers and graft them onto the COFs skeleton [98].

Due to the advantages of high thermal stability, high specific surface area, rigid topology, and good modifiability, COFs have great advantages in sensing, selective capture of pollutants, energy storage, separation, and catalysis, showing a broad development prospect of COFs [106]. In recent years, some research groups at home and abroad have conducted distinctive research on the applications of COFs, and COFs have been successfully applied on a large scale, becoming an important part of green chemistry [107].

## 6. Application of COFs for Detection of Antibiotics

COFs, due to their high stability, large specific surface area, and easy functionalization, can provide an alternative to easily detect antibiotics with high sensitivity, mainly in two approaches, namely, biosensors and adsorbents [108].

### 6.1. As Biosensors for Detection of Antibiotics

Biosensors have achieved vigorous and rapid development in recent decades because of their good selectivity, high sensitivity, fast analysis speed, low cost, and online continuous monitoring in complex systems, including environmental monitoring, health care, and food inspection [109]. COF-based biosensor materials show a high specific surface area and porous skeleton, which can provide a large surface interface for biomolecule immobilization, and in this way, the aptamer chain can not only be attached to the COF substrate by π–π stacking and hydrogen bonding but also be bound by the electrostatic interaction between the amino terminal functional group and the negatively charged aptamer, facilitating their applications in detecting antibiotics [110].

**Figure 8:**
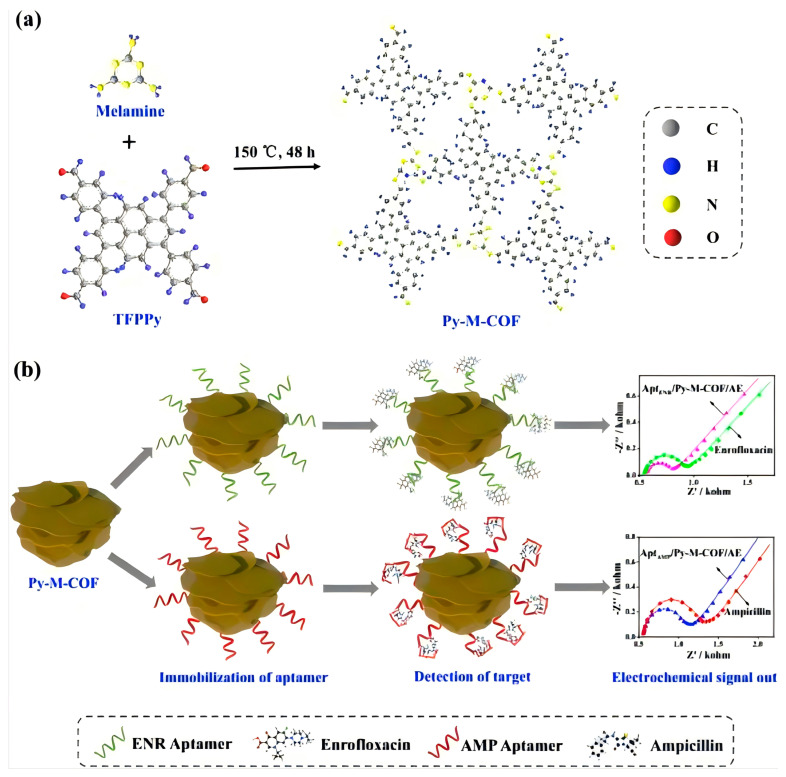
Scheme of electrochemical detection of enrofloxacin and ampicillin using COF-based aptasensors. Reproduced with permissions from Ref. [112]; Elsevier, copyright 2019.

Yao, D.’s work was related to a new Au nanocluster@ covalent–organic framework (Au@COF) sensor and applied to detect ultatrace small molecules, and the Au@COF sensor showed excellent sensitivity and good accuracy, proving the great potential of COF materials to be used as a sensor in the detection of biological samples [111]. Wang et al. condensed 1,3,6,8-tetra (4-formylphenyl) pyrene and melamine to synthesize an imine-based COFs material. This COF material was used as an electrochemical aptamer sensor for the detection of different kinds of antibiotics. The results showed that this method had a lower detection limit (6.07 and 0.04 fg mL^−1^ for enrofloxacin and ampicillin, respectively) and excellent sensing performance, which effectively extended the COFs as sensor materials to the sensitive detection of antibiotics [112]. Figure 8 shows the scheme of electrochemical detection of enrofloxacin and ampicillin using COF-based aptasensors.

Sun, Y. et al. used a simple one-pot strategy to effectively graft COFs onto carbon nanotubes and used them as electrical sensing materials to detect nitrofuran antibacterial agent furazolidone. As a network coating, COFs made the sensing materials have a high specific surface area (147.3 m^2^ g^−1^) and good sensing and separation properties (7.75 × 10^−8^ M when the detection ranged from 0.2 μM to 100 μM) [113].

Yang, Y. et al. successfully synthesized a highly sensitive SERS immunosensor based on COFs for the simultaneous detection of multiple foodborne pathogens. By using COF as a site to load non-bio-interference Raman signals and specific antibodies for the detection of foodborne pathogens, it showed strong non-interference characteristic Raman signals (2271 and 2113 cm^−1^ for two different foodborne pathogenic bacteria), and it could realize the simultaneous detection of two components. This COFs-based sensor provided a new idea for the detection of pathogenic bacteria [114].

Zhang, X. et al. prepared a new photoelectrochemical aptamer sensor based on 2D porphyrin COFs and used it for rapid, label-free detection of C-reactive protein. Due to the strong and rigid covalent bonds of COFs, the COF-based sensor had good conductivity and stability. In the experiment results, it was shown that the COF-based sensor had good analytical performance, such as high stability, sensitive response, good selectivity, and a wide linear range, making the COF-based sensor an ideal candidate for biological detection [115].

### 6.2. As the Adsorbent Materials for Pretreatment Technology

Pretreatment procedures are one of the most important steps in extraction methods, and the fast, simple, and automatic pretreatment technology not only saves time and labor but also reduces the error caused by different personnel’s operation and sample transfer [116]. So far, the common pretreatment methods include supercritical fluid extraction, solid-phase microextraction (SPME), solid-phase extraction (SPE), liquid-phase microextraction, membrane-separation technology, etc. [117,118].

**Figure 9:**
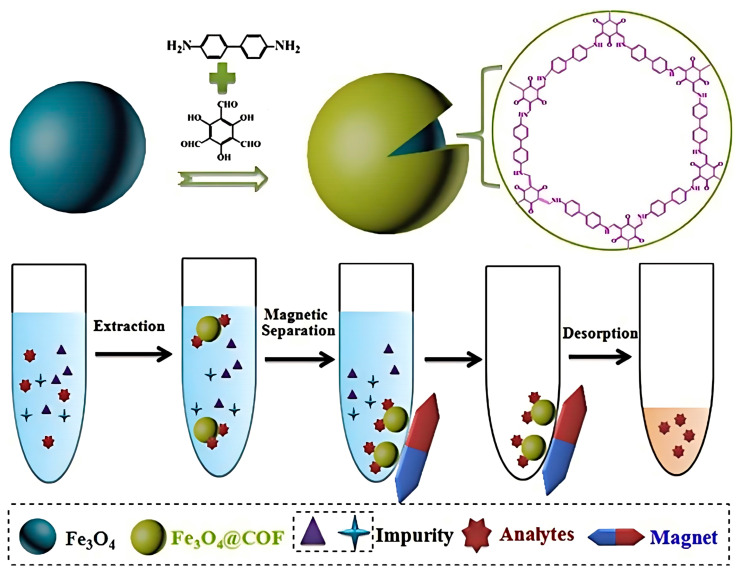
The fabrication process and application of magnetic COFs in MPE. Reproduced with permissions from Ref. [120]; Elsevier, copyright 2018.

In recent years, COF materials have received more and more attention in the field of sample processing; for example, COFs show obvious advantages in SPE, magnetic solid-phase extraction (MSPE), SPME, and chromatographic separation, with a high adsorption capacity and enrichment efficiency [119,120]. Figure 9 shows the fabrication process and application of magnetic COFs in MPE. This review mainly introduces the application of COF materials in SPE and SPME for the detection of antibiotics in water solutions.

#### 6.2.1. SPE

The SPE procedure uses a solid adsorbent to adsorb target compounds, separate them from the sample matrix and interfering compounds, and then elute or heat them with the eluent to achieve the purpose of separating and enriching target compounds [121]. A high-efficiency adsorbent is the key factor of SPE, which can improve the recovery rate and enrichment multiple and reduce the consumption of organic solvents, costs, etc. [122]. COFs not only have high crystallinity, excellent specific surface area, and high porosity but also have different pore sizes, which can be used as adsorbents for SPE to adsorb active ingredients efficiently and quickly [123].

Wang et al. used triazine-based COFs as the adsorbent of SPE and then used it in the separation of five sulfonamides and four tetracyclines. In the experiment, the triazine-based COFs showed a good extraction efficiency for the studied antibiotics (recoveries: 88.0–93.4%, and limit of detection (LOD): 0.031–0.55 μg L^−1^, and relative standard deviation (RSD): 2.5–4.8%), and the reusability was also tested for five cycles after absolute elution and balance of each cycle, proving the stability of adsorption characteristics. The successful application of triazine-based COFs to the separation of multiple antibiotics proved that the COF material was a promising antibiotic adsorbent [124].

**Figure 10:**
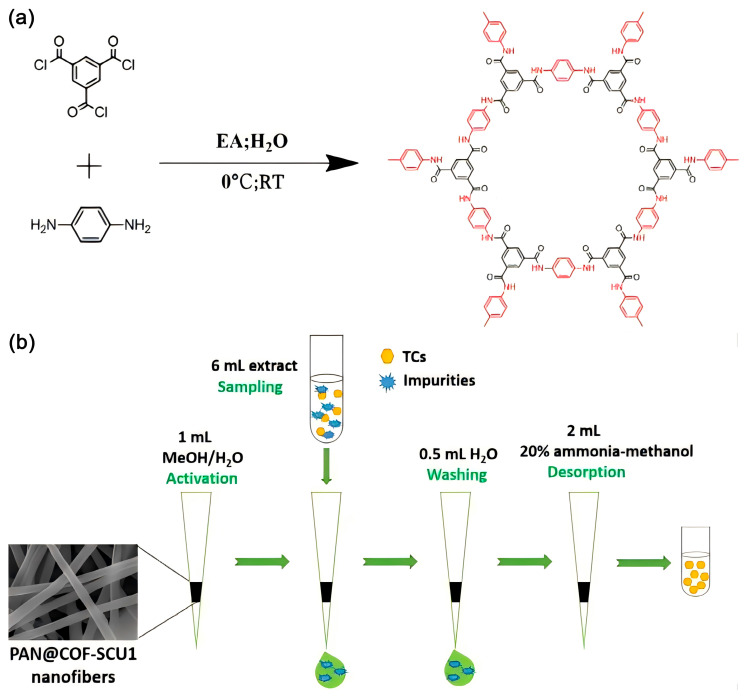
Schematic diagrams of preparation of COFs (**a**) and application in SPE procedure (**b**) for the extraction of tetracyclines. Reproduced with permissions from Ref. [125]; Elsevier, copyright 2020.

Wang et al. synthesized a new type of COF using a simple electrospinning method and used it as an adsorbent for SPE for enriching tetracycline antibiotics. The LOD (range from 0.6 to 3 ng mL^−1^), the limit of quantitation (LOQ) (range from 2 to 10 ng mL^−1^), and the recoveries decreased less than 30% after five consecutive cycles of SPE operation, exhibiting acceptable reproducibility and reusability. As an SPE adsorbent, the COFs have a fast adsorption rate and high adsorption performance, expanding the potential application of COFs in sample detection [125]. Figure 10 shows the schematic diagrams of the preparation of COFs (a) and application in SPE procedure (b) for the extraction of tetracyclines.

Wen et al. designed and synthesized a spherical tribenzodimethoxy dimethyl terephthalate-based COF and used it as an SPE adsorbent to extract polar sulfonamide antibiotics from water samples. The COF had rich adsorption sites and showed excellent adsorption performance for trace polar sulfonamides in water. The obtained COF had a low detection limit (0.5–1.0 ng L^−1^) and good repeatability and could be reused over 20 times without evident loss in the enrichment efficiency (76–87%). These results indicated that the COF was an important tool for the study of pollutant adsorption and was of great significance [126].

Wen et al. combined Fe_3_O_4_@COF with MSPE and used this method for the extraction and adsorption of fluoroquinolones. The LOD of this method for fluoroquinolones was 0.1–1.0 μg kg ^−1^ and had good linearity (R^2^ ≥ 0.9989), and the RSD values (3.8–7.7%) of inter-day precisions were repeated three different days, indicating good stability. The experimental results showed that the Fe_3_O_4_@COF in the detection of fluoroquinolones in complex liquid matrices had important application prospects [127].

#### 6.2.2. SPME

SPME was first proposed by Pawliszyn et al. in 1989, and its principle was to melt various cross-linked and bonded stationary phases on the mandrel in the syringe with an outer sleeve [128]. When using, the mandrel was pushed out and immersed in the crude sample solution, and the components to be measured were adsorbed on the mandrel, and then the mandrel of the probe was directly inserted into the sample inlet of the detector. Finally, the components to be measured were analyzed at the sample inlet and entered the instrument for analysis [129]. COFs as SPME adsorbents can obtain a high extraction and enrichment efficiency owing to their large specific surface area, suitable pore size, and functional groups on the surface [130].

The magnetic COFs were synthesized by Zhang et al., and they showed good adsorption efficiency (RSDs less than 5.1%), high recoveries (in the range of 87.0–113.8%), and recoveries of approximately 80% after six recycles, demonstrating good stability and reusability when used as SPME adsorbent to adsorb tetracycline. These studies have opened the door to the application of COFs in the field of chromatographic separation technology, which is very beneficial to the application and promotion of effective analysis technology of COFs in trace antibiotics in water samples [131].

**Figure 11:**
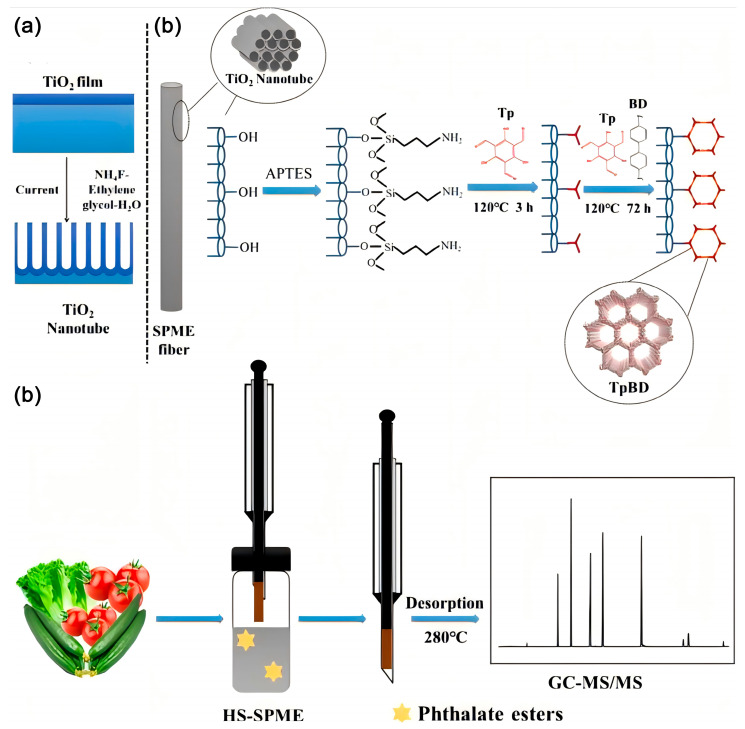
Schematic diagram of (**A**) electrochemical anodization on the surface of titanium wire, (**B**) preparation of TpBD-TiO2 SPME fiber, and (**C**) application of TpBDTiO2 fiber for HS-SPME and detection of phthalate esters. Reproduced with permissions from Ref. [132]; Elsevier, copyright 2020.

Yue et al. prepared COF by using 1,3,5-trimethylphloroglucinol and benzidine samples. This functionalized COF was used as the adsorbent in SPME to adsorb phthalates, and the COF fiber could be used repeatedly at least 150 times with a significantly low loss of extraction efficiency (<4.8%), limits of detection for phthalate esters (ranging from 0.001 to 0.430 μg/L), and enrichment factors (ranging from 226 to 2154). These research results strongly proved that COFs had a bright future in the adsorption of antibiotic organics [132]. Figure 11 shows the schematic diagram of the preparation of SPME COF-fiber and its application in the detection of phthalate esters in the SPME process.

Guo et al. combined 1,3,5-tris (4-formylphenyl) benzene and benzidine to prepare a COF fiber for the SPME of the selective identification and adsorption of polychlorinated biphenyls in aquatic products. The experimental results showed that the method had good recoveries (87.1–99.7%), a high enhancement factor (4471–7488), low detection limit (0.07–0.35 ng L^−1^), high accuracy (RSD < 100 ng L^−1^), and the peak areas of 100 ng L^−1^ had no significant changes after 180 extraction cycles. This work realized the detection of aromatic toxic organic pollutants in the extracted environmental samples of COFs and expanded the application of COFs in the adsorption field [133].

Ji et al. realized the application of the COF with dioxin linkage as an SPME coating to adsorb perfluoroalkyl substances in water. The designed COF-based SPME method showed high precision (RSD ≤ 7.9%), good linearity (0.01–1000 ng L^−1^) (R^2^ ≥ 0.9945), and low LOD (0.0020–0.0045 ng L^−1^), and the COF-coated fibers could be reused at least 20 times without the loss of extraction performance. These results show the prospect of the application of COFs in SPME and greatly encourage researchers to devote themselves to the application research of COFs in the field of extraction [134].

### 6.3. Comparison between COF-Based Materials and Other Materials for Detection of Antibiotics

By now, various kinds of materials have been studied for antibiotic detection, including MOFs, graphene, graphene oxide, quantum dots, MIPs, and other kinds of nanomaterials. However, using these materials for antibiotic detection exhibited some drawbacks; for example, the synthesis process for MIPs was complex and generated some toxic by-products [135]. Classical materials and methods that were used for the detection of antibiotics from water samples are presented in Table 2. In the table, it is shown that compared with other materials, COFs revealed better recovery, lower limits of detection, and RSD in the detection of antibiotics from water samples.

## 7. Adsorption Mechanism of Antibiotics by COFs

COFs have gained significant attention as a promising material for the adsorption of antibiotics. Lots of research has been conducted on their adsorption mechanisms of antibiotics, and the adsorption mechanism mainly includes the following aspects: porosity, pH value, and charge interaction; π–π stacking; hydrogen bonding; and hydrophobic interaction [152].

### 7.1. Porosity

COFs have high porosity and a large specific surface area, which enable them to act as carriers with adsorption activity. On the other hand, they reduce the aggregation of inorganic nanoparticles and introduce more adsorption sites to a greater extent. The characteristics of the corresponding structure greatly improve the adsorption performance of the composite materials and further efficient and sustainable adsorption [153].

Jiang et al. displayed the SO_3_H-anchored COFs with markedly high adsorption capacity of fluoroquinolone antibiotics (726 mg g^−1^, 531 mg g^−1^, and 9.5 mg g^−1^, respectively) due to the inerratic pore distribution [154]. Gendy et al. found that the specific surface area and porosity of COFs profoundly affected their adsorption capacity for organic pollutants, and this work laid a foundation for the subsequent development of COFs with better performance [155].

### 7.2. pH Value and Charge Interaction

The influence of the pH and charge interaction is mainly reflected in the electrostatic interaction force [156]. The pH value has an important influence on the adsorption reaction process, which affects the existence form of antibiotics in the water environment (charged or uncharged). The Zeta potential of the adsorbent can judge the positive and negative charges of the adsorbent under different pH values and then adsorb antibiotics through electrostatic effect [157].

Xu et al. investigated the influence of the pH on the adsorption capacity of TAPA-TFPB-COFs for quinolone antibiotics (QAs). In their study, to a certain extent, the adsorption performance increased with the increase in the pH value. The recoveries of QAs were found to be relatively high and stable with little change in the range of 5.0–9.0, which reflected the influence of the pH value on the adsorption efficiency [158].

### 7.3. π–π Interaction and Designability of Functional Group

COFs not only extend the π conjugated system to the entire three-dimensional structure but also expand the delocalization range of π electrons while inhibiting the π–π interaction between conjugated units. Therefore, COFs have high electron mobility and superior selectivity [159]. Moreover, COFs can introduce adsorption sites on the monomer by introducing charged groups or groups containing lone pair electrons on the monomer through monomer design and using their mutual use with the ions to be adsorbed to introduce the adsorption sites. The introduction of corresponding functional group ion groups on the polymerized COFs fixes COFs with a good adsorption capacity in the adsorption process [160].

Guan, S. et al. synthesized magnetic COFs and studied their adsorption of fluoroquinolone antibiotics by introducing specific functional groups. The adsorption recovery of the fluoroquinolones was found to be as high as 90.4 to 101.2%, with a low detection limit (0.05 to 0.20 μg/L), and the enrichment factor reached 115.5–127.3, which benefitted from the related functional groups [161].

## 8. Conclusions and Perspective

In recent years, COF has attracted more and more attention in the fields of gas storage, photoelectric, catalysis, sensors, etc. Especially, COFs have become a very popular and cutting-edge research topic, providing strong technical support for many fields and showing huge application potential.

Moreover, COFs have excellent application prospects in the detection of antibiotics from aqueous solutions due to their high and regular porosity, large specific surface area, adjustable pore size, and excellent structural stability. The COFs can better separate the target substance from the coexisting matrix interfering substance, achieve better purification and enrichment, and have the advantages of high detection sensitivity, good reproducibility, and low detection cost.

Despite the remarkable advances in COF-based applications that have been achieved, several challenges should still be addressed. Firstly, the synthetic optimization of a COF hybrid needs to aim at reducing the cost of COFs and increasing chemical compatibility, which is urgent for large-scale synthesis and practical use. Secondly, due to the complex activity of antibiotics, novel functional components of COFs should be developed, and it is necessary to systematically optimize the composition of COFs, such as organic ligands and coordination center ions, for efficient and sensitive separation.

## Figures and Tables

**Table 1:** The textural properties of some COFs.

Linkages Species	COFs	S_BET_ (m^2^ g^–1^)	Pore Volume (cm^3^ g^–1^)	Pore Size (nm)	Ref.
Boronate ester	COF-5	-	-	2.7	[52]
Imine	TpBD	885	-	2.3	[58]
Imine	CCOF-5	655	0.51	0.62	[59]
Amide	COF-6	613	0.42	0.59	[59]
Imine	Tx-COF-2	1137	-	1.51	[64]
Triazine	CTF-1	791		1.2	[53]
Triazine	CTF-Th	78	-	-	[54]
Triazine	Phen-CTF	358			[55]
Hydrazone	TFPT-COF	1.185		3.8	[66]
Hydrazone	BtaMth COF	723	0.46	1.41	[67]
Amide	COF-1	714	-	3.7	[73]
Azine	ACOF-1	1.176	-	1.1	[68]
Azine	Py-Azine COF	1.210	-	1.9	[69]
β-ketoenamines	Acridine COF	654	-	-	[70]

**Table 2:** Comparison of the present method for antibiotics detection from environmental water with other reported methods.

Materials	Detection Methods	Antibiotics Targets	Limits of Detection	Recovery (%)	Relative Standard Deviation (%)	Ref.
Zn(II)-MOF	Photoluminescence sensing	Aminoglycosides	37.6 ng L^−1^	97		[136]
Zr-MOF	Solid-phase extraction	Sulfonamides antibiotics	0.20 μg·mL^−1^	91–109.4	1.0–8.6	[137]
MIP	Sensor	Erythromycin	0.10 μg·mL^−1^	91–102		[138]
Graphene Quantum Dots	Fluorescent emission	Tetracycline	1.0 μg·L^−1^	85.3–103.3		[139]
MIP	Sensor	Amoxicillin	1.89 ng mL^−1^			[140]
MIP	Solid-phase extraction	β-lactam	9.56 μg·L^−1^	60–90		[141]
Graphene oxide hydrogel	Fluorescent biosensor	Oxytetracycline	25 μg·L^−1^			[142]
ZnO nanorods	Sensor	Trimethoprim	0.3 μg·mL^−1^	93.2–108		[143]
Nanomaterial	Sensor	Amoxicillin	50 ng L^−1^	97	3.3	[144]
Copper nanomaterials	Sensor	Amoxicillin	1.71 μg·mL^−1^	95	5	[145]
Quantum dot	Fluorescence sensor	Tetracycline	50 ng L^−1^	90.2–97.2	2.2–5.7	[146]
Quantum dots	Fluorescence sensor	Ofloxacin	0.3 ng mL^−1^	92–101	8	[147]
COFs		Enrofloxacin	0.05 ng mL^−1^	96.7–102.2	0.9–6.4	[148]
Fe_3_O_4_@COFs	Fluorescence sensor	Tetracycline	0.092 ng mL^−1^	96.4–103.7		[149]
Ba@COF	Fluorescence sensor	Rifampicin	0.03 μg·mL^−1^	75.20–123.46		[150]
COFs	Solid-phase extraction	Quinolone	0.02–0.06 ng∙L^−1^	68.2–104	<10%	[151]
